# Foundation Models in Cancer Pathology: Techniques, Applications, and Future Directions

**DOI:** 10.34133/research.1332

**Published:** 2026-06-26

**Authors:** Bo Zhang, Victor Yu Cui, Tong Wu, Guowei Su, Qinyi Huang, Bing Shang, Mei Kong, Weimiao Yu, Yuan Yuan, Huakang Tu

**Affiliations:** ^1^ Center of Clinical Big Data and Analytics of the Second Affiliated Hospital and the School of Public Health, Zhejiang University School of Medicine, Hangzhou 310058, China.; ^2^ Vanke School of Public Health, Tsinghua University, Beijing 100084, China.; ^3^ School of Public Health and Emergency Management, Southern University of Science and Technology, Shenzhen 518055, China.; ^4^ Department of Pathology, The First Affiliated Hospital of Zhejiang University School of Medicine, Hangzhou 310003, Zhejiang, China.; ^5^ Intelligent Digital and Molecular Pathology Lab, Bioinformatics Institute, A*STAR, Singapore 138671, Singapore.; ^6^ Department of Pathology, Yong Loo Lin School of Medicine, National University of Singapore, Singapore 138671, Singapore.; ^7^ Institute of Molecule and Cell Biology, Agency of Science Technology and Research (A*STAR), Singapore 138671, Singapore.; ^8^ School of Biological Science (SBS), Nanyang Technological University (NTU), Singapore 138671, Singapore.; ^9^ Department of Biochemistry, Yong Loo Lin School of Medicine (NUS Medicine), National University of Singapore, Singapore 138671, Singapore.; ^10^ Tumor Etiology and Screening Department of the Cancer Institute, and the Key Laboratory of Cancer Etiology and Prevention of Liaoning Education Department, the First Hospital of China Medical University, Shenyang 110001, China.; ^11^ Key Laboratory of Gastrointestinal Cancer Etiology and Prevention, Shenyang 110001, China.; ^12^ Institute of Digital and Intelligent Health, Zhejiang University, Hangzhou 310058, China.; ^13^ Zhejiang Key Laboratory of Intelligent Preventive Medicine,Hangzhou 310058, China.

## Abstract

Computational pathology enables scalable analysis of pathology images for cancer diagnosis and research, but conventional deep learning models remain constrained by annotation dependence and task-specific development. Recent advances in self-supervised learning, transformer-based architectures, and large-scale pretraining have given rise to computational pathology foundation models (CPathFMs), which are designed to learn transferable and reusable representations from diverse pathology data. This review first traces the development of CPathFMs by examining the data resources, backbone architectures, representation levels, input modalities, and pretraining strategies that shape their design. We then examine their applications across major cancer pathology tasks, including tumor detection, grading, and subtyping, molecular biomarker and gene expression prediction, prognostic assessment, tissue phenotyping, as well as multimodal retrieval and report generation. We further discuss key challenges that limit translation and deployment, including underrepresentation of normal tissues and benign lesions, limited generalization across domains and institutions, data overlap and benchmark contamination, task-centric evaluation and insufficiently standardized metrics, high training and infrastructure costs, and ethical concerns in model governance. Overall, CPathFMs mark an important shift from task-specific pathology artificial intelligence toward reusable representation learning. Their clinical value remains to be established through improved model performance and generalizability, more comprehensive and standardized evaluation frameworks, and prospective evidence of utility in real-world pathology workflows.

## Introduction

Pathology has long been central to cancer diagnosis and clinical decision-making, guiding tumor classification, treatment planning, and prognostic assessment. With the advent of digital slide scanners, glass slides can now be converted into high-resolution whole-slide images (WSIs), allowing tissue and cellular morphology to be analyzed computationally [1–4]. In routine practice, diagnosis still depends on pathologists’ expert visual assessment of tissue morphology, a process that can be affected by inter- and intraobserver variability [5–7]. Computational pathology integrates artificial intelligence (AI) with digitized pathological images to support more consistent and quantitative analysis of tissue and cellular morphology [8]. AI is now being explored as a useful tool in pathology, with potential value for cancer research and clinical practice [9–12].

**Fig. 1.**
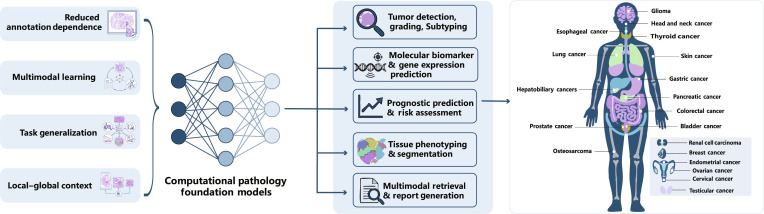
Computational pathology foundation models (CPathFMs) in cancer pathology: advantages, applications, and cancer coverage. The figure summarizes selected aspects of CPathFMs discussed in this review, including their potential advantages, representative application scope, and cancer types reported in the reviewed studies. The human body diagram indicates reviewed cancer sites rather than comprehensive clinical coverage or prospective validation.

Foundation models (FMs) have recently emerged in computational pathology as pretrained models that learn reusable representations from large-scale pathology datasets [13–16]. Their development is partly a response to practical challenges faced by earlier AI models, many of which were designed for specific tasks and depended heavily on expert annotations or task-specific labels, limiting their scalability and generalizability [17]. Moreover, because many earlier AI models were built within particular data or clinical settings, their performance may be sensitive to differences in staining, tissue processing, digitization, population demographics, and institutional data sources when applied beyond their development settings [18]. Obtaining such task-level supervision, can be time-consuming and costly to obtain, especially when pathologist review is needed or rare tumors are involved [19]. By training on large and diverse pathology datasets, including weakly labeled or unlabeled WSIs, FMs aim to learn representations that can be adapted to different downstream applications. Figure 1 provides a conceptual overview of computational pathology foundation models (CPathFMs), summarizing their key advantages, representative downstream applications, and the cancer sites represented in the reviewed studies.

Several recent reviews have examined CPathFMs from different perspectives. Some provide overviews of CPathFMs, covering representative models, datasets, and training strategies [15,16,20]. Others focus on more specific aspects, such as multimodal model design, adaptation and evaluation strategies, benchmark tasks, and taxonomy frameworks [21–23]. These works have helped clarify the technical landscape of CPathFMs. This review follows recent progress from model development to cancer pathology applications and translational challenges. The main contributions of this review are as follows:

• This review describes how computational pathology has evolved from task-specific models to FMs by summarizing the key technical components of CPathFMs and explaining their potential advantages in annotation efficiency, multimodal learning, task generalization, and local–global context modeling.
• This review organizes existing studies around major cancer pathology applications, including tumor detection, grading, and subtyping; molecular biomarker and gene expression prediction; prognostic prediction and risk assessment; tissue phenotyping and segmentation; and multimodal retrieval and report generation, linking technical advances to practical use.
• This review identifies unresolved challenges that may affect the development and translation of CPathFMs, including underrepresentation of normal tissues and benign lesions, cross-setting generalization, data overlap and benchmark contamination, clinical utility evaluation, training and operational costs, and ethical and regulatory governance. It further discusses future directions for improving robustness, evaluation, responsible model sharing, and workflow integration.

## Emergence of CPathFMs

Computational pathology has developed alongside successive advances in image analysis. Early studies relied mainly on handcrafted features and classical machine learning, whereas later convolutional neural network (CNN)-based methods substantially improved performance in tasks such as cancer detection and grading. Building on these developments, FMs aim to build task-agnostic foundation representations from large-scale data, which can then be adapted to different downstream applications. A common paradigm involves pretraining followed by fine-tuning, in which models are first pretrained without being optimized for a single predefined task, thereby learning generalized and domain-relevant representations, and are then adapted to specific downstream tasks with limited task-specific supervision [24].

Large-scale public pathology repositories are key resources for developing and evaluating pathology FMs. The Cancer Genome Atlas (TCGA) [25,26] has made a large collection of WSIs accessible across many tumor types and organ sites. The breadth and scale of these resources provide an important foundation for self-supervised representation learning. Other publicly available pathology-related datasets, including those from the Clinical Proteomic Tumor Analysis Consortium (CPTAC) with paired proteogenomic profiles [27], CAMELYON16/17 for breast cancer lymph-node metastasis detection [28,29], PANDA for prostate cancer grading [30], and Genotype-Tissue Expression (GTEx) for normal tissue gene-expression resources with associated tissue specimens [31], among others, have further expanded the available data ecosystem. In addition to public resources, many CPathFMs incorporate institution-specific or self-collected WSIs, which may help fill gaps in underrepresented tissue types, rare tumor subtypes, and staining variations, while improving data diversity and supporting robustness evaluation across centers.

**Fig. 2.**
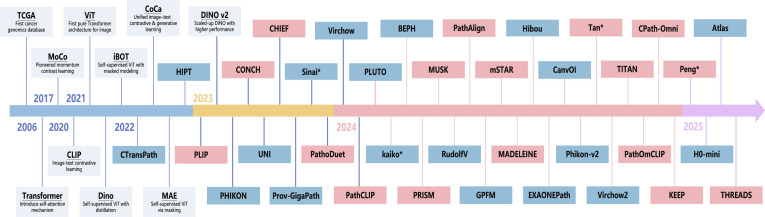
Timeline of key milestones in the development of computational pathology foundation models. Light gray boxes indicate fundamental technologies, blue boxes indicate unimodal models, and red boxes indicate multimodal models. Asterisks (*) indicate models for which no official name was provided in the original publication; these models were named using the first author’s name, research team, or affiliated institution. TCGA, The Cancer Genome Atlas; ViT, Vision Transformer; SSL, self-supervised learning; MAE, masked autoencoder; CLIP, contrastive language-image pretraining.

Alongside data expansion, advances in model architectures and learning algorithms have been equally critical. In 2017, the Transformer architecture introduced an attention-based framework that later influenced many medical image and multimodal learning models [32–34]. Building on this, the Vision Transformer (ViT) was introduced in 2020 as a representative pure transformer-based architecture for image analysis, providing a basis for global-context modeling in visual representation learning [33–35]. Subsequently, advances in large-scale pretraining methods, including self-supervised visual learning and multimodal alignment, substantially improved representation learning from unlabeled, weakly paired, or cross-modal data and laid the groundwork for the emergence of FMs [36–45]. From 2023 onward, research activity in the development of CPathFMs has accelerated, signaling the onset of a rapid growth phase in the field. Figure 2 summarizes these key milestones, including the release of critical architectures, learning paradigms, and landmark models.

## Model Design and Pretraining Strategies

**Fig. 3.**
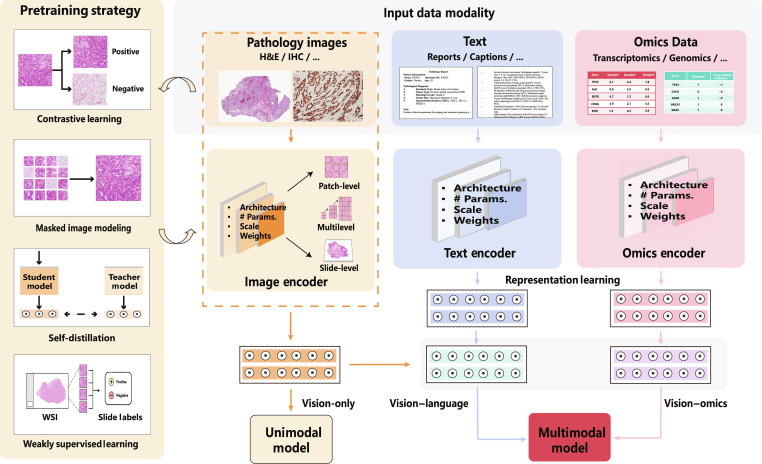
General development pipeline of computational pathology foundation models (CPathFMs). This figure summarizes the main components involved in developing CPathFMs. Pathology images are encoded into reusable visual representations at the patch level or slide level or across multiple representation levels. Text and omics data can be processed by modality-specific encoders and incorporated with pathology image representations through multimodal representation learning. The left panel summarizes representative pretraining strategies, including contrastive learning, masked image modeling, self-distillation, and weakly supervised learning. These strategies may be used alone or in combination to train vision-only, vision–language, or vision–omics models.

Figure 3 provides an overview of the general CPathFM development pipeline, from input modalities and encoder architectures to representation learning and pretraining strategies. Building on this framework, this section discusses key design choices in current CPathFMs, and representative models are summarized in Table 1.

**Table 1.** Summary of computational pathology foundation models

Models	Pretraining strategy	Backbone architecture ^a^	Representation level	Inputmodality ^b^	Pretraining data ^b^	Link	Evaluation scope	Key reported contribution
CTransPath [76]	Semantically relevant contrastive learning (SRCL) based on MoCo v3	P: CNN + Swin Transformer [28M]	Patch-level	Image-only	~15M patches from TCGA and PAIP WSIs (>30K WSIs; >25 anatomic sites and 32 cancer subtypes reported).	https://github.com/Xiyue-Wang/TransPath	5 Task types; patch retrieval/classification, WSI classification, mitosis detection, gland segmentation; 9 datasets.	**Semantic contrastive learning** Introduced SRCL to mine semantically related positive pairs and a CNN-Swin encoder for local–global histology features across patch and WSI downstream tasks.
HIPT [52]	DINO	P: ViT256/16	Multilevel	Image-only	10,678 WSIs across 33 cancer types; 408,218 4,096 × 4,096 regions; ~104M 256 × 256 patches.	https://github.com/mahmoodlab/HIPT	9 slide-level tasks; TCGA cancer subtyping and survival; hierarchical SSL benchmarks.	**Hierarchical gigapixel modeling** One of the first ViT-based hierarchical SSL frameworks for pathology, linking local morphology with region-level tissue context for slide classification and survival prediction.
		R: ViT4096/256						
		S: ViT-WSI/4096						
PLIP [62]	CLIP	P: ViT-B/32 [86M]	Patch-level	Image + text	OpenPath: 208,414 pathology image–text pairs curated from medical Twitter and other public sources.	https://github.com/PathologyFoundation/plip	4 external zero-shot datasets; linear probing plus image–text/image retrieval.	**Public image–text pretraining** Showed that publicly shared pathology image–caption data can support zero-shot classification and image/text retrieval.
		T: Transformer Layers [63M]						
Phikon [64]	iBOT	P: ViT-B/16 [86M]	Patch-level	Image-only	43,374,634 histology tiles from 6,093 TCGA slides across 16 cancer types.	https://github.com/owkin/HistoSSLscaling	17 tasks; patch and WSI classification/segmentation across 7 cancer indications.	**In-domain masked pretraining** Demonstrated that pathology-specific iBOT pretraining improves many patch- and slide-level tasks over ImageNet-pretrained and MoCo v2 baselines.
						https://huggingface.co/owkin/phikon		
CONCH [49]	P: iBOT	P: ViT-B/16 [86M]	Patch-level	Image + caption	1.17M human histopathology image–caption pairs for visual–language pretraining; image encoder was pretrained on ~16M 256 × 256 image tiles.	https://github.com/mahmoodlab/CONCH	14 benchmarks; zero-shot classification, retrieval, segmentation, and captioning.	**Patch-level vision–language generalization** Provided a strong pathology VLM for zero-shot classification, retrieval, segmentation, captioning, and label-efficient downstream learning.
	T: GPT-style	T: GPT-style [86M]						
	CM: CoCa	D: GPT-style				https://huggingface.co/MahmoodLab/CONCH		
UNI [46]	DINOv2	P: ViT-L/16 [307M]	Patch-level	Image-only	Mass-100K: >100M tissue patches from 100,426 H&E diagnostic WSIs across 20 major tissue types.	https://github.com/mahmoodlab/UNI/	34 tasks; ROI classification/segmentation/retrieval, slide classification, and pan-cancer tasks.	**Data/model scaling** Established large-scale DINOv2 scaling trends in pathology and reported strong results across 34 diverse tasks, including few-shot slide classification.
						https://huggingface.co/MahmoodLab/UNI		
CHIEF [50]	Stage 1: Self-supervised Patch-level Pretraining	P: CTransPath	Slide-level	Image-only	15M unlabeled tiles for tile pretraining; 60,530 WSIs spanning 19 anatomical sites for WSI-level weakly supervised pretraining.	https://github.com/hms-dbmi/chief	32 independent datasets; cancer detection/origin, genomic-profile prediction, and survival.	**Slide-level weak supervision** Combined tile pretraining and WSI-level weak supervision to support cancer detection, tumor-origin identification, mutation prediction, and prognosis across independent cohorts.
		S: Attention-based feature fusion with WSI-level contrastive learning						
	Stage 2: Weakly Supervised WSI-level Pretraining	T: Text Encoder of CLIP						
Prov-GigaPath [55]	P: DINOv2	P: ViT-G/14 [1.1B]	Slide-level	Image-only	Prov-Path: 1.3B 256 × 256 tiles from 171,189 WSIs from >30,000 patients across 31 major tissue types.	https://github.com/prov-gigapath/prov-gigapath	26 tasks; cancer subtyping and pathomics; Providence and TCGA cohorts.	**Open whole-slide scaling** Released an open whole-slide FM trained on real-world health-system data; reported state-of-the-art performance on 25 of 26 benchmark tasks.
	S: MAE	S: LongNet [125M]				https://huggingface.co/prov-gigapath/prov-gigapath		
Sinai ^d^ [114]	DINO	P: ViT-S [86M]	Patch-level	Image-only	>3B images from 423,563 digital microscopy slides from 88,035 cases and 76,794 patients.	https://github.com/sinai-computational-pathology/OPAL/tree/main/SinaiPathologyFoundationModels	6 clinical tasks; breast, GI, and lung cohorts; 2-institution evaluation.	**Institution-scale SSL comparison** Demonstrated large academic-health-system SSL pretraining and compared DINO with MAE, with DINO generalizing better in the reported benchmark.
	MAE	P: ViT-L [303M]						
PathoDuet [61]	MoCo v3	P: ViT-B/16 [86M]	Patch-level	Image-only, multistain (H&E + IHC)	H&E: 13,166,437 patches and 1,623,258 regions from ~11K TCGA diagnostic FFPE WSIs; cross-stain data from HyReCo and BCI paired H&E/IHC slides.	https://github.com/openmedlab/PathoDuet	H&E/IHC tasks; patch subtyping, WSI classification, marker expression, and tumor identification.	**Cross-scale and cross-stain transfer** Explicitly models H&E cross-scale relationships and transfers structural knowledge to IHC, introducing a paired H&E/IHC foundation-model framework.
Virchow [47]	DINOv2	P: ViT-H/14 [632M]	Patch-level	Image-only	~2B 224 × 224 tiles from ~1.5M H&E WSIs from MSKCC.	https://github.com/Paige-AI/paige-ml-sdk	Pan-cancer and rare-cancer detection; biomarker prediction; tile-level probes.	**Million-slide scaling** Scaled pathology FM training to million-slide data and enabled pan-cancer detection, rare-cancer detection, and biomarker prediction with strong label efficiency.
						https://huggingface.co/paige-ai/Virchow		
PathCLIP (PathAsst) [112]	CLIP-style	P/T: PathCLIP (OpenAI CLIP-based)	Patch-level	Image + caption	PathCap: 207K high-quality pathology image–caption pairs; PathAsst further used ~180K instruction-following samples.	https://huggingface.co/jamessyx/pathclip	4 zero-shot classification datasets; cross-modal retrieval; PathVQA for PathAsst.	**Pathology CLIP assistant foundation** Improved pathology retrieval and zero-shot classification and served as the visual encoder for the PathAsst multimodal generative assistant.
	Multimodal instruction tuning	LM: Vicuna-13B						
PLUTO [77]	DINOv2 ^c^	P: FlexiViT-S/(8,16,32) [22M]	Patch-level	Image-only	195M image tiles from ~158K WSIs across >50 data sources, multiple stains, scanners, magnifications, and disease areas.	/	Multiscale benchmarks; slide classification, tile classification, and instance segmentation.	**Lightweight multiresolution deployment** Designed a compact FlexiViT backbone that supports segmentation, tile classification, and slide prediction with far fewer parameters than many large FMs.
Kaiko ^d^ [92]	DINO	P: ViT-S/(8,16) [22M], ViT-B/(8,16) [86M], ViT-L/14 [307M]	Patch-level	Image-only	TCGA WSIs (~29K WSIs); models trained and evaluated with a scalable online-patching pipeline.	https://github.com/kaiko-ai/towards_large_pathology_fms	eva benchmarks; linear probes, segmentation, TP53, and magnification/data-scale ablations.	**Open training/evaluation infrastructure** Provided an open training pipeline, hyperparameter/data-scale analyses, and eva for standardized pathology FM evaluation.
	DINOv2					https://huggingface.co/1aurent/vit_base_patch16_224.kaiko_ai_towards_large_pathology_fms		
BEPH [65]	BEiTv2	ViT-B/16 [86M]	Patch-level	Image-only	11,774,353 patches from 11,760 TCGA pathology images across 32 cancer types.	https://github.com/Zhcyoung/BEPH	11 tasks; patch recognition, WSI subtype classification, survival, and limited-data tests.	**Lightweight masked-image modeling** Introduced a deployable MIM-based pathology FM evaluated for patch recognition, WSI classification, and survival prediction under limited-label settings.
PRISM [54]	CoCa-style	P: Virchow ViT-H/14 [632M]	Slide-level	Image + pathology report text	587,196 WSIs from 195,344 specimens with associated clinical reports.	https://huggingface.co/paige-ai/Prism	Cancer detection/subtyping, biomarker prediction, report generation; zero-shot and fine-tuning.	**Generative specimen-level VLM** Produces slide/specimen embeddings and report text, improving zero-shot cancer detection/subtyping and label-efficient biomarker prediction.
		S: Perceiver Net						
		T/D: BioGPT [345M]						
MUSK [67]	Stage 1: P: BEiT-3-style T: BERT-style	P: ViT/16	Patch-level	Image + text	50M pathology image patches from 32,898 slides; 1B pathology-related text tokens; 1M image–text pairs for alignment.	https://github.com/lilab-stanford/MUSK	23 benchmarks; retrieval, VQA, classification, biomarker prediction, prognosis, and treatment response.	**Unified multimodal pretraining** Uses both unpaired and paired image/text data for multimodal pretraining and evaluates broadly across patch, slide, and precision-oncology outcome tasks.
		T: Transformer Layers						
	Stage 2: CM: Contrastive learning	CM: shared self-attention				https://huggingface.co/xiangjx/musk		
RudolfV [115]	DINOv2 ^c^	ViT-L/14 [304M]	Patch-level	Image-only, multistain	~1.2B tiles from 133,998 slides/34,103 cases across >15 labs, 58 tissue types, and 129 staining modalities.	/	12 benchmarks/31 datasets; TME profiling, IHC biomarkers, rare-case search, and robustness.	**Pathologist-guided data curation** Incorporated pathologist domain knowledge into data curation and sampling, improving robustness for tumor microenvironment profiling, biomarker evaluation, and reference search.
PATHALIGN [79]	P: Masked Siamese Networks	P: ViT-S S: Q-Former of BLIP-2	Slide-level	Image + pathology report text	Over 350,000 WSI-diagnostic text pairs, plus TCGA diagnostic WSIs for cancer enrichment.	/	Pathologist-rated WSI-text retrieval/generation; 4 WSI classification tasks and triage.	**WSI-text alignment** Learns language-aligned WSI embeddings for retrieval, classification, triage, and WSI-level text generation; pathologists rated most generated text clinically acceptable.
	S/CM: BLIP-2-style							
GPFM [75]	DINOv2 ^c^ + expert distillation	ViT-L/14 [307M]	Patch-level	Image-only student model; multimodal expert knowledge is distilled into the image encoder	~190M images from ~86,000 public H&E WSIs across 34 major tissue types.	https://github.com/birkhoffkiki/GPFM	39 Tasks across 6 task types; external validation on 7 tasks; generalization benchmark.	**Knowledge distillation across FMs** Distills complementary strengths from multiple expert FMs into a single encoder and reported the best average rank across 39 downstream tasks.
						https://huggingface.co/majiabo/GPFM		
mSTAR [68]	Stage 1: CLIP-style	P: ViT-L	Multilevel	Image + pathology report text + RNA-seq	26,169 slide-level modality pairs from 10,275 TCGA patients across 32 cancer types; 22K modality pairs used for pretraining.	https://github.com/Innse/mSTAR	12 Diagnostic/molecular tasks; 9 survival tasks; few-/zero-shot classification and reports.	**Multimodal slide-to-patch transfer** Injects WSI-level image-report-RNA-seq knowledge into pathology FMs, broadening patch-only features with slide-level multimodal context.
		S: TransMIL						
		T: BioBert- Basev1.2 [44]				https://huggingface.co/Wangyh/mSTAR		
	Stage 2: Self-Taught Training	RNA-seq: scBERT-style						
MADELEINE [116]	P: GOT	P: CONCH	Slide-level	Image-only, multistain (H&E + IHC/special stains)	Breast cohort: 4,211 WSIs across 5 stains; kidney-transplant cohort: 12,070 WSIs across 4 stains.	https://github.com/mahmoodlab/MADELEINE	21 Tasks; few-shot/full classification, survival, IHC quantification; 7,299 WSIs.	**Cross-stain slide representation** Uses matched H&E/IHC/special stains as informative views for task-agnostic slide representation, supporting morphology, molecular, prognostic, and IHC-quantification tasks.
	S: InfoNCE					https://huggingface.co/MahmoodLab/madeleine		
	CM: INTRA	S: Multi Head-MIL						
Hibou [117]	DINOv2	ViT-B/14	Patch-level	Image-only, multistain and cytology included in training data	Private dataset with 936,441 H&E slides, 202,464 non-H&E slides, and 2,676 cytology slides; Hibou-B used 512M clean patches and Hibou-L used 1.2B clean patches.	https://github.com/HistAI/hibou	Patch and slide public benchmarks; TCGA BRCA/NSCLC/RCC subtyping; PanNuke segmentation.	**Open multistain ViT family** Released an open family of DINOv2 pathology ViTs trained on a large diverse private dataset and benchmarked on patch- and slide-level tasks.
						https://huggingface.co/histai/hibou-L		
						https://huggingface.co/histai/cellvit-hibou-l		
		ViT-L/14				https://huggingface.co/histai/hibou-b		
EXAONEPath [118]	DINO	ViT-B/16 [86M]	Patch-level	Image-only	285,153,903 patches from 34,795 WSIs.	https://github.com/LG-AI-EXAONE/EXAONEPath	6 Patch-level classification tasks; linear evaluation and stain-normalization analysis.	**Stain-normalized efficient training** Identified WSI-specific feature collapse and used stain normalization during FM training to improve efficiency and generalization on 6 patch-level tasks.
						https://huggingface.co/LGAI-EXAONE/EXAONEPath		
CanvOI [119]	DINOv2	ViT-g/10 [1.1B]	Patch-level	Image-only	70,217,688 tiles from 632,608 tissue samples, mostly H&E, from >100 international source sites and >40 cancer types.	/	5 Slide-level oncology tasks; BRACS, HunCRC, NSCLC, OOD, and label-scarcity tests.	**Tile-size/patch-size scaling** Explored an alternative scaling axis by increasing tile size and reducing ViT patch size, with reported AUC gains over comparator FMs, especially in low-label regimes.
Phikon-v2 [48]	DINOv2	ViT-L/16 [307M]	Patch-level	Image-only	~460M pathology tiles from 58,359 WSIs across >100 public cohorts and >30 cancer sites/normal tissues.	https://huggingface.co/owkin/phikon-v2	8 Slide-level biomarker/subtyping tasks; external validation cohorts and ensembling.	**Public biomarker-oriented scaling** Expanded Phikon to a ViT-L trained on 460M public pathology tiles, achieving competitive performance with leading proprietary foundation models.
Tan ^d^ (PMPRG) [81]	Stage 1: DINO	P: VGG16	Multilevel	Image + pathology report text	In-house multiorgan dataset of 7,422 WSIs with clinical reports; evaluated on colon and kidney cases.		Patient-level report generation; diagnosis/grade classification; NLG and clinical-efficacy metrics.	**Clinical report generation** Proposed patient-level multiorgan pathology report generation from variable numbers of WSIs, with multiscale regional attention and tag-guided explainability.
		R/S: MR-ViT						
	Stage 2: Supervised Learning	LM: GPT-2-style medical LM						
Virchow2 [120]	DINOv2 ^c^	P: ViT-H/14 [632M], ViT-G/14 [1.9B], ViT-S/14 [22M]	Patch-level	Image-only, multistain/mixed magnification	All 3 models trained from 3.1M histopathology WSIs; reported tile counts include ~1.7B for Virchow2, 2B for Virchow2G, and ~0.9-1B for Virchow2G Mini.	https://huggingface.co/paige-ai/Virchow2	ID/OOD tile benchmarks plus HEST; scaling, mixed-magnification, and distillation analyses.	**Domain-specific scaling and distillation** Combines data diversity, pathology-specific training changes, model-scale growth, and distillation to achieve strong tile-level results while offering a compact mini model.
TITAN [56]	Stage 1: iBOT	P: CONCHv1.5	Slide-level	Image + caption	335,645 WSIs; 423,122 ROI-caption pairs generated by PathChat; 182,862 WSI-report pairs.	https://github.com/mahmoodlab/TITAN	Few-/zero-shot classification, rare-cancer retrieval, cross-modal retrieval, prognosis, and reports.	**Whole-slide vision–language modeling** Extends pathology VLMs to WSI representations for zero-shot classification, rare-cancer retrieval, cross-modal retrieval, prognosis, and report generation.
		S: ViT slide encoder						
	Stage 2–3: CoCa-style	T: CONCHv1.5 text encoders				https://huggingface.co/MahmoodLab/TITAN		
		D: CONCHv1.5 multimodal decoders						
PathOmCLIP [121]	CLIP	S: GigaPath	Multilevel	Image + spatial transcriptomics	HEST benchmarking 10x Visium dataset with paired 224 × 224 H&E patches and matched spatial gene-expression spots across 5 tumor types.	/	HEST 10x Visium; 5 tumor types; spatial gene-expression prediction and ablations.	**Spatial-omics alignment** Combines pathology and single-cell foundation models with local-neighborhood modeling to improve spatial gene-expression prediction from histology.
		P: LocalTransformer						
		Spatial transcriptomics: scFoundation						
CPath-Omni [80]	DINOv2 & CLIP	P: Virchow2 & CLIP-L	Patch-level + Slide-level	Image + caption/instructions	CPath-CLIP: 700,145 image–caption pairs; CPath-Omni: 351,871 patch-level instruction samples, 5,850 WSI reports, 33,830 WSI instruction samples, plus patch-level instruction mixing.	/	7 Tasks across 42 datasets; patch/WSI VQA, classification, captioning, and visual referring.	**Unified pathology LMM** Reported a 15B-parameter pathology LMM unifying patch and WSI analysis for classification, VQA, captioning, and visual referring, with strong results across many datasets.
		S: SlideParser						
		T: Qwen2-1.5B						
		LM: Qwen2.5-14B						
KEEP [78]	Knowledge-enhanced CLIP-style	P: ViT-L/16	Patch-level	Image + text + disease knowledge graph	Disease KG with 11,454 human diseases and 139,143 disease attributes; public pathology image–text data reorganized into 143K semantic groups.	https://huggingface.co/Astaxanthin/KEEP	18 WSI diagnostic benchmarks; segmentation, detection, subtyping; tile retrieval/classification.	**Knowledge-enhanced zero-shot VLM** Injects hierarchical disease knowledge into VLM training, improving zero-shot cancer detection, segmentation, and rare cancer subtyping.
		T: PubMedBERT						
						https://huggingface.co/Astaxanthin/KEEP		
Atlas [122]	DINOv2	ViT-H/14 [632M]	Patch-level	Image-only, multistain/multimagnification	1.2M WSIs from Mayo Clinic and Charité; 3.4B tiles extracted and ~520M sampled for training; >70 tissue/organ types, >100 stains, 7 scanner types.	/	21 Public benchmarks; eva/HEST plus TME, morphology, molecular and cancer-typing tasks.	**Multi-institution data diversity** Showed that broad institutional, stain, organ, and scanner diversity can yield strong average performance across public benchmarks at intermediate model/data scale.
THREADS [60]	P: CoCa	P: CONCHv1.5	Slide-level	Image + transcriptomics + genomics	MBTG-47K: 47,171 H&E tissue sections paired with molecular profiles; 26.6K RNA-seq samples and 20.1K genomic-variant samples reported.	https://github.com/mahmoodlab/patho-bench	54 Oncology tasks; subtyping, mutation, IHC, treatment response, and survival.	**Molecularly supervised WSI FM** Uses transcriptomic and genomic supervision to train a WSI FM evaluated on 54 oncology tasks, improving label efficiency and rare-event prediction relative to WSI baselines.
	S: ABMIL	S: ABMIL [11M]						
	CM: CLIP	RNA-seq: scGPT				https://huggingface.co/datasets/MahmoodLab/Patho-Bench		
		DNA variants: MLP						
Peng ^d^ [123]	Continual multimodal learning with pseudo target generation module (PTGM), instruction-based knowledge distillation, and modality-specific MM-LoRA	P: ViT	Patch-level	Image + clinical/pathology text + RNA-seq	TCGA cohorts with text, WSI image features, and bulk RNA-seq; experiments reported on UCEC, LUAD, LGG, BRCA, and BLCA.	/	TCGA prognosis across 5 cancers; continual multimodal adaptation and forgetting tests.	**Continual multimodal prognosis learning** Addresses continual adaptation of multimodal cancer-prognosis models by mitigating catastrophic forgetting within and across modalities.
		T: BERT-style						
		RNA-seq: BulkRNABert						
H0-mini [124]	DINO & iBOT	ViT-B/14 [86M]	Patch-level	Image-only	43M 224 × 224 tiles at 20× magnification from 6,093 TCGA slides across 16 cancer types.	https://huggingface.co/bioptimus/H0-mini	HEST/EVA and robustness benchmarks; PLISM and multiscanner/stain tests.	**Compact distillation** Demonstrates that distillation can produce a compact pathology FM with competitive HEST/EVA benchmark performance and improved robustness to stain/scanner variation.

P, patch-level; R, region-level; S, slide-level. In “Pretraining strategy”, these prefixes denote the level of the training objective; in “Backbone architecture”, they denote the corresponding encoder or model component. T, text encoder; CM, cross-modal module, component, or objective; D, decoder; LM, language model; ST, spatial transcriptomics; RNA-seq, RNA sequencing; ViT, Vision Transformer; WSI, whole-slide image; CNN, convolutional neural network; TCGA, The Cancer Genome Atlas; VQA, visual question answering; H&E, hematoxylin and eosin; IHC, immunohistochemistry; MIM, masked image modeling; AUC, area under the curve

^a^ For simplicity, we have streamlined some expressions. For example, “/8” denotes a patch size of 8 ×8 pixels, and “/ (8,16)” represents “/8” and “/16”, respectively.

^b^ Unless otherwise specified, pathology images refer to H&E-stained WSIs or tiles.

^c^ Indicates modifications or extensions made to the original framework.

^d^ When a study does not provide an official model name, we use the first author’s name, research team, or affiliated institution followed by an asterisk (^d^) to denote the model.

### Backbone architectures

ViT-based backbones [33] are widely adopted in current CPathFMs [46–50]. Compared with CNNs, ViTs rely less on local convolutional inductive biases and can model broader spatial relationships through self-attention, making them suitable for capturing both cellular morphology and tissue architecture [51,52]. At the slide level, many CPathFMs further extend this design by aggregating tile embeddings with hierarchical transformers, slide transformers, or long-context transformer architectures [53–56], enabling broader modeling of WSI-level spatial organization. CNN–transformer hybrids and hierarchical variants such as Swin Transformers [57] are also used in some image encoders, particularly when local feature extraction, computational efficiency, or limited data are important considerations. In multimodal CPathFMs, the vision backbone is often combined with modality-specific encoders, such as transformer-based text encoders or molecular encoders, to enable alignment between pathology image representations and textual or omics information.

### Representation levels

CPathFMs can be broadly categorized according to the level at which pathology representations are learned and used, including patch-level, multilevel, and slide-level models.

**Patch-level models.** Many CPathFMs use small tissue patches, tiles, or regions of interest (ROIs) as the basic input unit. This patch-based strategy was already common in earlier weakly supervised WSI methods, such as CLAM [58] and TransMIL [59]. WSIs are tiled into small tissue patches (e.g., 256 × 256 pixels), each of which is processed independently by a ViT-based or other visual backbone to produce local feature embeddings. For slide-level applications, these embeddings may be summarized by attention-based multiple-instance learning (MIL) or pooling modules, or further processed by slide-level transformers or dedicated WSI encoders to generate slide-level representations or predictions. This modular design separates local feature extraction from slide-level modeling, making patch embeddings reusable across tasks and cohorts. However, because patches are encoded independently, spatial relationships among them are not explicitly modeled within the encoder. Consequently, their ability to capture coherent tissue structures depends strongly on the downstream aggregation or WSI-level modeling strategy.

**Multilevel models.** These models organize pathology information across more than 1 spatial scale or representation level, rather than relying on a single patch- or slide-level representation. A common form is the hierarchical model, in which lower-level features are progressively aggregated into higher-level representations. For example, HIPT [52] uses hierarchical self-supervised learning to encode both 256 × 256 patches and larger 4,096 × 4,096 regions before deriving slide-level representations. By retaining intermediate representations before final WSI aggregation, multilevel designs can preserve mesoscale spatial evidence and may support more localized interpretation than approaches that directly compress all tile features into a single global WSI embedding.

**Slide-level models.** These models typically begin with patch embeddings, but the pretrained and reusable representation is defined at the WSI level. For example, PRISM [54] aggregates Virchow tile embeddings into slide-level embeddings with clinical report supervision, while Prov-GigaPath [55] uses a tile encoder followed by a LongNet-style slide encoder to model long sequences of WSI tiles. TITAN [56] and THREADS [60] further expand slide-level modeling through vision–language or vision–omics pretraining. Because they must integrate thousands to tens of thousands of tile features from each WSI, slide-level models usually require a dedicated whole-slide modeling step beyond independent patch encoding. This step can increase graphics processing unit (GPU) memory use, training time, and the need for large slide-level datasets.

### Input modalities

Based on input modalities, CPathFMs can be broadly categorized into 3 groups: vision-only, vision–language, and vision–omics models. Vision-only models use digitized pathology images as the primary input and remain the dominant form of CPathFMs, partly because routine WSIs provide large amounts of unlabeled morphology data suitable for large-scale self-supervised representation learning. Vision–language models incorporate textual information, such as pathology reports, captions, diagnostic labels, or instructions, to align pathology image representations with semantic concepts. This alignment supports tasks such as zero-shot classification, text-based retrieval, visual question answering (VQA), and report-related applications. Vision–omics models connect pathology images with genomic, transcriptomic, or spatial transcriptomic profiles. In these models, molecular information can guide representation learning and support tissue annotation, gene-expression prediction, biomarker inference, and biologically informed slide-level modeling.

### Pretraining strategies

CPathFMs are pretrained through a range of learning strategies that differ in their supervision signals, input modalities, and representation levels. Most pathology image encoders are first trained with self-supervised objectives, including contrastive learning, masked image modeling (MIM), and self-distillation, which allow models to learn reusable visual features from large collections of unlabeled patches or regions. Multimodal pretraining extends this idea by using text, molecular profiles, or other data types to guide, align, or integrate pathology image representations, often through contrastive, matching-based, generative, or modality-guided objectives. In addition, weakly supervised learning, commonly formulated as MIL, is used to learn slide- or patient-level representations from coarse diagnostic, molecular, or outcome labels. These categories are therefore not mutually exclusive but together describe the major routes through which CPathFMs acquire transferable representations.

**Contrastive learning and cross-modal alignment.** Contrastive learning trains models by defining positive and negative relationships between samples. Positive pairs are pulled closer in the representation space, whereas unrelated or mismatched samples are pushed apart. In pathology, such pairings may include 2 augmented views of the same patch, multiscale views from the same tissue region, paired hematoxylin and eosin (H&E) and immunohistochemistry (IHC) images, or image–text pairs used for cross-modal alignment. Representative frameworks include visual contrastive methods such as MoCo [36], image–text contrastive methods such as contrastive language-image pretraining (CLIP) [37], and contrastive-captioning methods such as CoCa [41]. For example, PathoDuet [61] introduces cross-scale positioning and cross-stain transfer as pretext tasks to learn representations from H&E and IHC images. This design supports representation learning across different magnifications and stains and improves performance in downstream patch- and WSI-level tasks. For vision–language modeling, PLIP [62] uses a CLIP-style dual encoder trained on pathology image–text pairs to align patch features with free-text pathology descriptions, enabling zero-shot classification and text-based retrieval across multiple organs and cohorts.

**MIM.** MIM trains models by hiding part of an image and asking the model to reconstruct or predict the missing content [63]. In pathology, many diagnostic patterns depend not only on isolated cells but also on surrounding tissue architecture, such as glandular organization, stromal context, and tumor boundaries. Representative frameworks such as MAE [42] and BEiT v2 [45] have shown that masking-based objectives can scale with ViT backbones and large unlabeled image datasets, and this idea has since been adapted to pathology image pretraining [64]. For example, BEPH [65] adopts BEiT v2 [45] for patch-level pretraining on more than 11 million TCGA tiles and then reuses the ViT backbone as a general-purpose feature extractor for both slide-level cancer detection and survival prediction across a wide range of cancer types.

**Self-distillation.** Self-distillation is another label-free strategy for learning pathology image features. It typically trains a model to produce consistent representations from different augmented views of the same tissue patch, such as different crops or color variations. In many implementations, this is achieved through a teacher–student design: The student network learns from a more stable teacher network, which provides target representations during pretraining. Because this process does not require manual annotations, it can be scaled to large collections of unlabeled pathology images. It is particularly useful in histopathology, where the same tissue pattern may appear differently because of staining variation, scanning conditions, magnification, or tissue orientation. Representative frameworks include DINO [40] and DINOv2 [44], as well as iBOT [38], which combines teacher–student self-distillation with masked token prediction. For example, DINOv2-style self-distillation has been adopted by models such as UNI [46] and Virchow [47] to pretrain pathology image encoders, producing transferable patch-level features for diverse downstream pathology tasks.

**Weakly supervised learning.** Weakly supervised learning is commonly used when detailed region- or pixel-level annotations are unavailable but slide-, specimen-, or patient-level labels are available. In computational pathology, this setting is often formulated as MIL, where a WSI is represented as a bag of patch or region embeddings and the model learns to aggregate them into a slide-level representation or prediction [17,58,66]. In CPathFMs, this strategy complements patch-level self-supervised pretraining by using coarse diagnostic, molecular, or outcome labels to learn higher-level representations across the whole slide or across multiple slides from the same patient. It reduces the need for dense expert annotation, but the learned representations may be affected by label noise, sampling bias, and uncertainty about which regions drive the slide-level label.

**Multimodal representation learning.** Multimodal pretraining is not limited to the contrastive alignment described above. In CPathFMs, additional modalities may be used through cross-modal integration, generative objectives, instruction tuning, knowledge distillation, or modality-guided supervision, even when a clearly named fusion module is not present. For example, MUSK [67] uses shared self-attention blocks to jointly model image and text representations, while mSTAR [68] incorporates pathology slides, reports, and RNA sequencing data within a unified pretraining framework. Other models use multimodal information mainly as training supervision or generation targets, such as PRISM [54], which leverages clinical report text for slide-level pretraining and report generation, and THREADS [60], which uses transcriptomic and genomic profiles to guide WSI-level representation learning. These examples show that multimodal CPathFMs differ not only in the modalities they use but also in whether cross-modal information is aligned, integrated, generated, distilled, or used as supervision during pretraining.

## Potential Advantages of CPathFMs

### Reduced dependency on annotated data

A major expected advantage of CPathFMs is their reduced reliance on dense expert annotations. By learning from large collections of unlabeled or weakly labeled WSIs, CPathFMs can provide reusable representations for downstream task adaptation. This is particularly relevant for rare cancer subtypes and diagnostically challenging cases, where labeled data are inherently scarce. For example, Phikon-v2 [48], based on the DINOv2 [44] framework, was trained on hundreds of millions of image patches and evaluated across multiple downstream tasks, illustrating how large-scale pretraining can provide reusable features for subsequent task adaptation. However, current evidence suggests that downstream performance still often depends on substantial task-specific annotated datasets, particularly for high-stakes diagnostic applications. In practice, model performance tends to improve with increasing amounts of labeled data, and many studies therefore rely on as much annotated data as available during downstream training. As a result, the extent to which FMs reduce annotation requirements remains difficult to quantify. Whether this paradigm ultimately translates into reduced annotation burden in real-world clinical settings remains unclear.

### Enhanced multimodal representation learning

Traditional pathology AI models have largely been developed around image-based inputs, whereas clinical diagnosis and cancer characterization often rely on multiple sources of information, including morphology, pathology reports, and molecular profiles. Increasingly, CPathFMs are moving toward multimodal representation learning, in which heterogeneous data sources such as images, reports, and molecular profiles can be jointly modeled or aligned to capture complementary clinical and biological information. For example, PLIP [62] aligns pathology image representations with textual descriptions, enabling tasks such as text-based retrieval and classification. Multimodal learning has also been used to combine WSIs with molecular profiles for pan-cancer analysis and outcome prediction [69]. Among such approaches, mSTAR [68] integrates pathology, pathology reports, and transcriptomic data to support molecular subtyping and survival prediction. However, current evidence is still largely derived from specific datasets and experimental settings, and multimodal gains require further validation across institutions, patient populations, and clinical scenarios.

### Improved multitask generalization and scalability

A key advantage of CPathFMs is their potential reuse across diverse downstream applications. Because they learn shared morphology representations that are not limited to a single diagnostic label, cancer type, or end point, these models can be adapted to different tasks using strategies such as linear probing, fine-tuning, MIL, or multimodal prompting. For example, UNI [46], pretrained on large-scale pathology datasets, demonstrates strong performance across a wide range of classification tasks, while multimodal models such as CONCH [49] support applications such as tumor subtyping, image–text retrieval, and vision–language tasks. These studies suggest that CPathFMs can serve as reusable backbones across tasks and may reduce the need to build separate models for each application. However, downstream performance often still depends on task-specific adaptation, and consistent generalization across tasks, datasets, and clinical settings has not yet been fully established [70]. The lack of appropriate evaluation frameworks further makes it difficult to assess this advantage, and its practical impact remains uncertain.

### Integrated local and global context analysis

Pathology interpretation is not limited to fine-grained cellular morphology, such as nuclear atypia, but also depends on broader tissue architecture and spatial organization, including glandular patterns, stromal arrangement, tumor–stroma interactions, and immune microenvironments. One potential advantage of CPathFMs is that large-scale pretraining on WSI-derived data may help models learn local morphological features together with broader tissue context, rather than treating them only as isolated patch-level signals. This is particularly important because many clinically relevant patterns emerge from the spatial arrangement of cells and tissue structures, not from single patches alone. Architecturally, this capacity is often supported by hierarchical or transformer-based designs that connect patch-level, region-level, and slide-level representations. For instance, HIPT [52] models information across multiple spatial scales by combining patch-level and region-level representations, whereas Prov-GigaPath [55] extends this idea to whole-slide modeling by capturing spatial dependencies across WSI tiles. In this way, CPathFMs provide a framework for integrating cellular morphology, tissue architecture, and slide-level organization into representations that better reflect both local and whole-slide context.

## Clinical and Research Applications

Computational pathology has supported a broad spectrum of applications in cancer research and clinical practice. Earlier deep learning approaches mainly improved performance in specific tasks, whereas CPathFMs broaden this scope by providing reusable representations for diverse diagnostic, molecular, prognostic, and multimodal pathology applications. In this section, we highlight 5 key application domains. Although these applications have not yet been prospectively validated in real-world clinical practice, existing studies provide evidence of their potential value across cancer pathology tasks.

### Tumor detection, grading, and subtyping

**Fig. 4.**
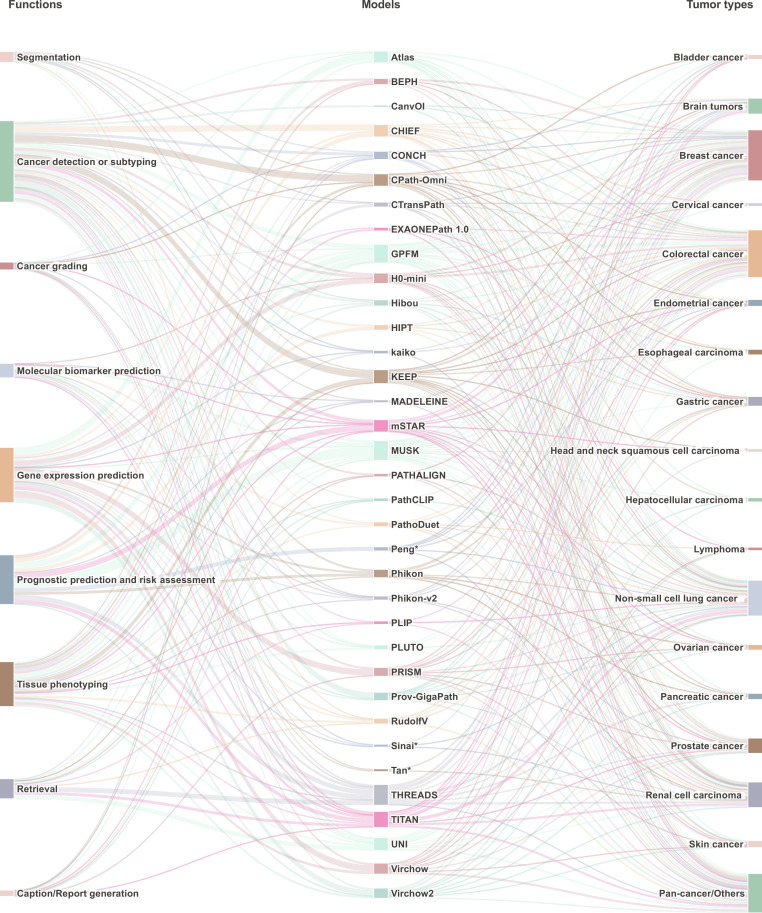
Functional landscape of computational pathology foundation models across cancer types and tasks. This Sankey-style diagram maps the relationships between functional tasks (left), foundation models (middle), and cancer types (right). Line thickness and node size represent frequency of association, not quantitative superiority of performance. For unnamed models, we adopt the convention of citing them using the first author’s name, research team, or affiliated institution followed by an asterisk.

Tumor detection, grading, and subtyping represent core applications of computational pathology, underpinning routine diagnostic workflows and subsequent clinical decision-making. Figure 4 shows that these tasks constitute the most extensively explored application area for CPathFMs. Among the models reviewed in this review, more than 30 models have been applied across a relatively broad range of cancer types, including major solid tumors such as breast, lung, renal, prostate, and colorectal cancer. Other clinically important cancers, including bladder, cervical, skin, and head and neck cancers, have been studied in only a limited number of works. Across these studies, CPathFMs have shown promising performance in selected tumor detection, grading, and subtyping tasks. In selected settings, leading models have reported area under the receiver operating characteristic curve values near 0.95 for pan-cancer or specimen-level cancer detection, as well as high top-k accuracy in multiclass organ–tumor classification tasks [46,47,49,50,54].

However, these findings should be interpreted in the context of real-world diagnostic workflows. In routine pathology practice, diagnosis, particularly for rare or diagnostically challenging tumors, often requires integration of H&E morphology with clinical context, IHC, and molecular testing. These considerations also highlight an important limitation of morphology-based inference alone and motivate increasing interest in integrating pathological features with molecular information, as discussed in the following section.

### Molecular biomarker and gene expression prediction

Molecular biomarkers and gene expression profiles play an important role in cancer prognosis and treatment but typically require sequencing assays or IHC [71], which can be costly, time-consuming, and not always available in routine practice. These limitations have motivated efforts to explore the extent to which molecular information can be inferred from pathological images [72,73]. Existing studies suggest that CPathFMs can support downstream inference of selected molecular biomarkers and gene-expression-related end points from pathological images.

As illustrated in Fig. 4, more than 20 models have been applied to molecular biomarker and gene expression prediction across several cancer types, including breast cancer, non-small cell lung cancer, and colorectal cancer. The most commonly investigated end points include clinically actionable biomarkers such as HER2 status, microsatellite instability, immunohistochemical marker status, and recurrent gene mutations, as well as broader transcriptomic or spatial gene-expression profiles. For selected molecular end points, leading models have reported moderate-to-strong predictive performance, with area under the receiver operating characteristic curve values commonly reaching the 0.80 to 0.90 range and, in some tasks, exceeding 0.90 [47,54,60,68]. Although these approaches are not intended to replace established molecular testing in clinical practice, they may still provide complementary value as scalable tools for preliminary assessment, case prioritization, or application within resource-limited settings.

### Prognostic prediction and risk assessment

Prognostic prediction and risk assessment are important applications in computational pathology, aiming to stratify patients based on survival risk and guide treatment decisions [74]. Unlike diagnostic classification, prognostic prediction depends on both histopathological features and reliable clinical-outcome data, including survival, follow-up, treatment, and patient-level variables, which are often incomplete or inconsistently collected across cohorts. Prognostic applications remain less common among the reviewed studies, with approximately 12 CPathFMs identified, primarily in common cancers such as breast cancer, non-small cell lung cancer, renal cell carcinoma, and colorectal cancer (Fig. 4).

Reported results suggest that CPathFMs can provide meaningful prognostic signals, with leading models reaching C-index values around 0.7 to 0.75 in held-out or pan-cancer settings [50,67,68,75]. Current studies are largely confined to a small number of well-studied cancers, with relatively little exploration in other tumor types. In addition, there is substantial variation in end points, cohort composition, and evaluation strategies across studies, making results difficult to compare directly. Broader validation is therefore needed before prognostic applications can be reliably compared or translated into clinical use.

### Tissue phenotyping and segmentation

Tissue phenotyping aims to characterize the composition and spatial organization of the tumor microenvironment, including immune infiltration, stromal components, and necrotic regions, which provide important information for tumor grading and subtyping, prognostic assessment, and treatment response evaluation. In computational pathology studies, such characterization is commonly achieved through segmentation of tissue regions or cellular components on pathological images.

According to Fig. 4, tissue phenotyping and segmentation have been explored by approximately 26 CPathFMs across multiple cancer types, particularly in breast and colorectal cancers. Representative applications include mitosis detection, gland segmentation, cancer-region segmentation, and zero-shot tissue segmentation. Reported results suggest strong performance in selected well-defined tasks, such as gland segmentation with Dice scores around 0.9 [76,77]. By contrast, zero-shot segmentation has shown lower performance in representative settings, with Dice scores around 0.6 [49,78]. Current applications remain unevenly distributed across cancer types, with limited exploration in many tumor settings. Tissue phenotyping and segmentation remain evolving application areas, where more standardized benchmarks and broader validation are needed.

### Multimodal retrieval and report generation

Multimodal retrieval and report generation aim to support case-based reasoning and structured reporting by integrating pathology images with textual information. These applications include image-to-text and text-to-image retrieval, similar-case search, pathology VQA, and preliminary report generation. In practice, they could help retrieve similar cases and assist in generating preliminary report drafts or case summaries.

As summarized in Fig. 4, a relatively limited number of CPathFMs have been applied to multimodal retrieval and report generation, with about 11 models for retrieval and 9 for report generation across cancer types such as colorectal, breast, and non-small cell lung cancers. Existing studies have reported top-k retrieval performance ranging from approximately 35% to 75% [49,56,62,67,79], open-ended WSI VQA accuracy around 67% [80], and report-generation scores around 0.6 to 0.7 using conventional text-overlap metrics such as METEOR or ROUGE-L [81]. These results suggest measurable but task-dependent progress, although such metrics do not necessarily reflect diagnostic correctness or clinical usefulness.

At present, multimodal retrieval and report generation remain at an early stage. Existing studies are largely retrospective and task-specific, with limited evidence on how these tools affect reporting efficiency, diagnostic consistency, or pathologist–AI interaction in real-world workflows. Their clinical value therefore remains to be established through prospective studies that assess model performance, interface design, workflow integration, and use in routine pathology settings [82–84].

## Challenges in Translation and Deployment

Despite the growing flexibility and transferability enabled by large-scale pretraining, CPathFMs still face several barriers before they can be reliably translated into clinical practice. These barriers arise not only from model performance itself but also from how models are evaluated, reproduced, implemented, and governed in real-world pathology settings.

### Underrepresentation of normal tissues and benign lesions

Current CPathFMs are often developed from cancer-enriched datasets and optimized around cancer-centered tasks [85]. This data and task orientation tends to emphasize malignant morphology, while normal tissues, reactive changes, inflammation, benign proliferative lesions, and benign mimics of malignancy receive less systematic coverage and less explicit attention. As a result, learned representations may be effective for overt cancer-related patterns but less capable of modeling the broader nonmalignant spectrum needed for diagnostic exclusion and differential diagnosis. This limitation may weaken the model’s ability to distinguish malignancy from benign mimics, account for background tissue context, and avoid overemphasizing cancer-associated features in ambiguous cases. In addition, attention- and MIL-based architectures may further reinforce this tendency by focusing on highly discriminative malignant regions at the expense of subtler benign or contextual patterns [86].

### Limited generalization across domains and institutions

Even when CPathFMs are pretrained on large, multi-institutional, or multidomain datasets, generalization across real-world clinical settings remains inconsistent. Such diverse pretraining is expected to improve robustness to heterogeneity across institutions and domains, but empirical studies continue to report performance variability under distributional shifts caused by differences in staining protocols, scanner hardware, tissue preparation, and institutional workflows [87–90]. These findings suggest that large-scale pretraining can improve cross-domain robustness to some extent but does not fully mitigate domain shift in real-world deployment. Robust and transferable generalization therefore remains an open challenge for CPathFMs in clinical practice.

### Data overlap and benchmark contamination

The extensive reuse of public datasets raises concerns about data overlap and benchmark contamination in current CPathFM evaluation. Many CPathFMs are pretrained on large public repositories such as TCGA and are subsequently evaluated on TCGA-derived cohorts or benchmarks that may partially overlap with pretraining data. Although this does not necessarily constitute data leakage in the strict supervised-learning sense, prior exposure to the same repositories, institutional patterns, cancer-type distributions, or potentially overlapping slides may reduce the independence of evaluation cohorts [18,91,92]. As a result, reported performance may overestimate true generalization ability, particularly when benchmarks do not clearly exclude pretraining–evaluation overlap or provide transparent data provenance.

### Task-centric evaluation and lack of standardized metrics

Current evaluation of CPathFMs remains largely task-centric, with model quality commonly inferred from performance across multiple downstream tasks involving different cancer types and molecular end points. Broader task coverage and consistently higher performance are often interpreted as evidence of stronger general-purpose representation learning. However, whether good performance across different cancer types, end points, and task settings, or aggregation across heterogeneous tasks, truly reflects the generalization capacity of CPathFMs remains unclear. The lack of standardized evaluation frameworks, together with substantial differences in datasets, task definitions, and experimental protocols, makes fair comparison across models difficult. Although recent studies have begun to develop benchmark frameworks for CPathFMs [70,93–95], most evaluations still rely on conventional task-based settings and provide limited evidence on clinical utility. In addition, evaluation metrics are still largely derived from traditional task-specific deep learning, focusing on area under the curve, sensitivity, and specificity. While these metrics are necessary for clinical validation, they do not fully capture the broader value of CPathFMs, such as reducing annotation burden, supporting label-efficient adaptation, enabling flexible transfer across tasks, and remaining computationally feasible for deployment. Future evaluation frameworks should therefore incorporate not only predictive performance but also data efficiency, adaptation flexibility, computational cost, and clinical workflow relevance [96–98].

### High training and infrastructure costs

The training cost of CPathFMs remains an important feasibility issue, as large-scale pretraining requires extensive WSI datasets and substantial computing infrastructure. Prov-GigaPath provides a useful quantitative example: It was pretrained on 1.38 billion 256 × 256 pathology tiles from 171,189 WSIs across more than 30,000 patients, and its preprocessing pipeline used up to 200 computing nodes, each with 32 central processing unit cores and 256-GB RAM, for approximately 157 h [55]. Slide-level pretraining further required 16 nodes with 4 80-GB A100 GPUs per node for approximately 2 d, corresponding to 3,072 A100 GPU-hours [55]. These figures indicate that the major computational burden lies in large-scale data preparation, tile extraction, storage, and distributed pretraining, which may be difficult to reproduce in routine pathology laboratories or smaller research settings. By contrast, inference may be relatively efficient once the model has been trained; Prov-GigaPath reported an average inference time of 0.7 s per WSI under its optimized setting [55]. However, direct comparison with conventional CNN pipelines remains difficult because hardware settings, preprocessing procedures, tile sampling strategies, and evaluation workflows vary across studies. More transparent reporting of preprocessing time, storage requirements, GPU hours, and per-slide inference time under clearly specified hardware settings would help readers better assess the practical feasibility of CPathFMs.

### Ethical concerns in model governance

Beyond widely recognized concerns in medical AI, such as data privacy, bias in data and algorithms, and limited interpretability [99,100], CPathFMs introduce additional ethical challenges. A central issue is the uncertainty surrounding large-scale pretraining data. These models are typically trained on aggregated datasets drawn from multiple institutions and sources, for which the exact composition, provenance, and consent conditions may be incompletely documented [101,102]. This raises questions about informed consent, secondary data use, and traceability, particularly given that WSIs are often linked to sensitive clinical information. At the same time, the aggregation of cross-institutional data introduces further governance complexity, as differences in regulatory frameworks, data ownership, and consent policies can complicate compliance and accountability, especially in multicenter or cross-border settings [103].

## Future Directions

### Strengthening morphological representation

Future CPathFMs should be developed to recognize a broader range of pathological patterns encountered in routine practice. Current models are often evaluated on tumor-centered tasks, such as cancer detection, grading, subtyping, molecular prediction, and prognosis, where performance may be driven mainly by highly discriminative malignant regions. However, reliable clinical use also requires models to distinguish cancer from normal tissue, benign lesions, inflammatory or reactive changes, precursor lesions, and borderline cases. Future studies should therefore examine how model design, sampling strategies, pretraining objectives, and slide-level aggregation affect performance in these challenging nonmalignant and borderline scenarios.

### Evaluating translational readiness

Future CPathFMs should be evaluated not only by benchmark performance but also by their readiness for clinical or research translation. Such evaluation should place greater emphasis on robustness under conditions that resemble routine pathology practice. Slides from different institutions may vary in staining quality, scanner type, tissue processing, specimen type, tumor subtype, and case mix, which are not always fully captured by standard benchmark datasets. Future studies could therefore assess model performance across external cohorts, low-quality slides, staining variations, rare subtypes, and diagnostically difficult cases to better understand how stable these models are under real-world conditions. In addition, translational evaluation should examine whether model outputs are interpretable, whether results are reproducible, and whether the model can be integrated into existing workflows for the intended clinical or research task.

### Defining the scope of CPathFMs

A key question for future CPathFM development is how to define the appropriate scope of different models, including broadly applicable pan-cancer models and more cancer-specific models. Pan-cancer CPathFMs provide reusable representations across diverse tumor types and remain valuable for general-purpose feature extraction. However, cancer-specific CPathFMs may offer advantages when the goal is to support well-defined clinical pathways within a specific disease context, such as lesion stratification, grading, subtyping, biomarker prescreening, risk assessment, or quality control. Recent studies have begun to explore this direction by leveraging large-scale pretraining within a single cancer type and adapting representations to specific downstream tasks [104–106]. Future work should clarify when disease-specific pretraining provides added value over a general-purpose FM and how validation should be matched to the model’s clinical claim.

### Sharing models responsibly

Because CPathFMs are intended to serve as reusable backbones across multiple downstream tasks, their value depends not only on performance reported in the original study, but also on whether they can be reliably reused, evaluated, and adapted by others. Future sharing practices should more clearly specify both the scope of release and the conditions of use [102]. Open weights provide an important starting point for reuse, but reproducible and comparable evaluation often requires implementation code, preprocessing details, input settings, and evaluation protocols. Released models should also state whether they are restricted to research use, noncommercial use, clinical validation studies, or commercial development. A more sustainable sharing model should balance openness, reproducibility, intellectual property protection, data governance, and appropriate downstream use.

### Integrating multiple CPathFMs

As more CPathFMs become available, future research should move beyond comparing individual models and explore how their complementary strengths can be integrated. Recent studies have begun to investigate this direction by combining or coordinating multiple CPathFMs at the feature, representation, prediction, or ensemble level [107–111]. Different CPathFMs may capture different aspects of pathology because they vary in pretraining data, model architecture, learning objectives, image scale, and modality design. Instead of relying on a single “best” backbone, multi-FM strategies could combine features from several models, use adapter or meta-encoder modules to combine or harmonize representations, or distill complementary information into a more efficient model. However, integration should not be assumed to improve performance by default. Future studies should test whether multi-FM integration provides consistent gains over strong single-model baselines and whether the added computational cost and complexity are justified for the intended application.

### Embedding CPathFMs in diagnostic workflows

Future CPathFMs should be developed with greater attention to integration into pathology workflows. In routine practice, pathologists need more than slide-level predictions; they need interpretable information that supports case review, differential diagnosis, quality control, and reporting. Recent multimodal pathology assistants, such as PathAsst [112] and PathChat [113], illustrate early efforts to combine pathology image analysis with language-based interaction, enabling users to ask pathology-related questions and receive image-informed responses that may support case review and explanation. Although still at an early stage, these systems point toward a shift from static prediction models to interactive decision–support tools. Future studies should examine how model-highlighted regions, uncertainty estimates, morphological evidence, and natural-language explanations can be integrated into digital pathology viewers and reporting workflows, while ensuring that CPathFMs support rather than replace expert judgment. Prospective workflow studies should further assess whether such tools improve diagnostic efficiency, consistency, and decision-making in real-world practice.

## Conclusion

CPathFMs represent an important shift in computational pathology from task-specific model development toward reusable representation learning across cancer pathology tasks. Their value lies not only in improving individual downstream performance but also in reducing dependence on task-specific annotations, supporting multimodal representation learning, enabling reuse across multiple downstream tasks, and integrating local morphological features with broader tissue and slide-level context. By doing so, they provide a shared modeling framework that can connect morphology with diagnostic, molecular, prognostic, and multimodal information. However, current evidence does not yet support viewing these models as clinically mature systems. Persistent challenges, including underrepresentation of normal tissues and benign lesions, limited generalization across domains and institutions, data overlap and benchmark contamination, task-centric evaluation and lack of standardized metrics, high training and infrastructure costs, and ethical concerns in model governance, indicate that CPathFM development remains an emerging paradigm rather than a source of ready-to-deploy clinical solutions. Future work should therefore place greater emphasis on clinically meaningful validation, broader morphological coverage, clearer model scope, transparent data provenance, reproducible and responsible model sharing, and workflow-compatible human–AI collaboration. With continued progress in these areas, CPathFMs may gradually develop from general-purpose pathology backbones into reliable tools that support cancer pathology research and, eventually, selected clinical applications.
